# A metabolic conspiracy drives anti-tumorigenic macrophages

**DOI:** 10.1093/lifemeta/load009

**Published:** 2023-03-11

**Authors:** Na Li, Tiffany Horng

**Affiliations:** School of Life Sciences and Technology, ShanghaiTech University, Shanghai 201210, China; School of Life Sciences and Technology, ShanghaiTech University, Shanghai 201210, China

In the tumor microenvironment, activation of macrophages through CD40 ligation promotes tumor restriction and is being considered clinically. The underpinning metabolic basis is not known and was addressed in a new study by Ho and colleagues.

Macrophage activation (or polarization) to distinct cellular states—that are associated with the acquisition of new cellular activities—is fueled by metabolic reprogramming. Intercepting such metabolic reprogramming can disrupt appropriate macrophage activation, thus there is increasing interest in targeting cellular metabolism to alter macrophage activities in a variety of pathophysiological settings [1]. In a recent study published in *Nature Immunology* [2], Ho and colleagues examined the metabolic basis of CD40-mediated macrophage activation. CD40 is a cell surface receptor of the TNFR superfamily, and its ligation (by its ligand CD40L, which is expressed on activated helper T cells) can activate macrophages to produce higher levels of pro-inflammatory cytokines. In the context of the tumor microenvironment (TME), ligation of CD40 using agonist antibodies activates tumor associated macrophages (TAMs) to a pro-inflammatory, anti-tumorigenic phenotype that restricts tumor growth [3]. Thus, anti-CD40 agonist antibodies are being considered in early phase clinical trials. The metabolic basis of CD40-mediated macrophage activation is not known, and was the central question asked by Ho and colleagues.

The authors first implicated mitochondrial oxidative metabolism in the enhanced inflammatory gene expression of CD40 activated macrophages. In an *in vitro* model where anti-CD40 agonist antibodies drove macrophage activation, oxidative metabolism was augmented, while pharmacological block of electron transport chain (ETC) activity attenuated the enhanced inflammatory gene expression. Early studies indicated that during macrophage activation, the induction of transcriptional programs that specified activation to a distinct cellular state is supported by gene-specific histone acetylation [4], while more recent studies indicated that metabolism can modulate such histone acetylation. Specifically, acetyl coenzyme A (Ac-CoA), the metabolic substrate for histone acetylation, appears to be limiting, such that regulation of Ac-CoA production could affect histone acetylation at the inducible genes that specify macrophage activation [5, 6]. In particular, Ac-CoA production in activated macrophages can be promoted by augmenting expression and/or activity of ATP citrate lyase (ACLY), an enzyme that produces a nuclear-cytoplasmic pool of Ac-CoA using as its substrate citrate, which is derived from the TCA cycle [4–6]. In parallel, Ac-CoA production can be fueled by increasing oxidative metabolism, thus augmenting substrate availability for ACLY [5, 6]. Indeed, Ho and colleagues found that in CD40 activated macrophages, ACLY expression was necessary to support the increase in inflammatory gene expression. Furthermore, ACLY deletion in the hematopoietic compartment was sufficient to abrogate the ability of anti-CD40 agonist antibodies to restrict tumor growth, which correlated with reduced inflammatory gene expression in TAMs [2].

**Figure 1:**
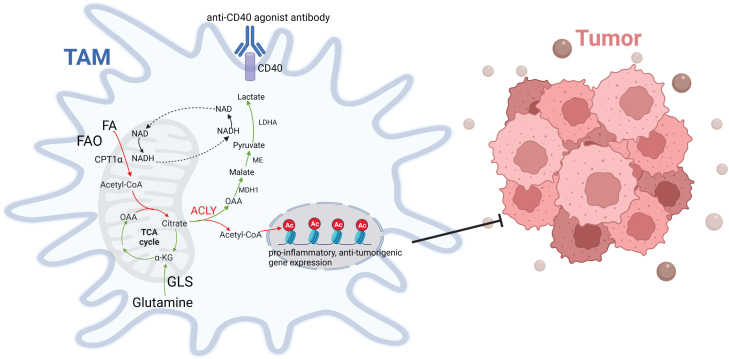
The metabolic pathway in which fatty acids and glutamine work together to promote pro-inflammatory and anti-tumorigenic activities in CD40-activated tumor associated macrophages.

If increased oxidative metabolism was supporting ACLY-dependent inflammatory gene expression in CD40-activated macrophages, what were the relevant carbon substrates? Using media deficient in or supplemented with various carbon substrates, isotope tracing experiments, and genetic knockouts of key metabolic enzymes, the authors uncovered that an unexpected metabolic pathway drives increased substrate delivery to ACLY. Specifically, when CD40 activation was triggered in glucose deficient media, glutamine oxidation appeared to be a major source of oxaloacetate (OAA), while fatty acid oxidation appeared to be a major source of Ac-CoA, and the two substrates condense to form the ACLY substrate citrate in the first step of the TCA cycle. In the cytosol, ACLY activity cleaves citrate into OAA and Ac-CoA, and in the CD40-activated macrophages, such Ac-CoA appeared to be used for histone acetylation at inflammatory genes, as discussed above, while OAA appeared to be converted to lactate, via a malate dehydrogenase 1 (MDH1)-malic enzyme (ME)-lactate dehydrogenase (LDH) axis (Fig. 1). Although in other settings, ACLY-generated OAA can return to the TCA cycle (via MDH1-mediated conversion to malate, which can be imported into the TCA cycle followed by conversion back to OAA) to promote further citrate production (thus driving additional rounds of cytosolic Ac-CoA production), such recycling of OAA was not engaged in the CD40-activated macrophages. Rather, the macrophages shunt OAA towards production of lactate, which was presumably released extracellularly. Such a shunt was needed to fuel inflammatory gene expression, because knockout of MDH1, ME, and LDH all attenuated inflammatory gene expression. The authors proposed that the OAA to lactate shunt may have a role in maintaining redox balance to support the induction of inflammatory genes. In support of this notion, LDH-deficient macrophages had a lower NAD/NADH ratio, while supplementation with nicotinamide riboside (NR), a precursor for NAD, normalized such ratio and partially restored inflammatory gene expression. Perhaps, a high NAD/NADH balance enabled high rates of substrate oxidation to provide a steady supply of carbon substrates for ACLY-dependent histone acetylation and inflammatory gene induction.

The metabolic pathway described in this study contrasts with that engaged in “classically” activated macrophages, in which glucose and glutamine oxidation fuels carbon substrate availability for histone acetylation to support the induction of genetic programs that coordinate antimicrobial immunity [6–8]. It also differs from that employed by the interleukin-4 activated macrophages that orchestrate tissue repair, which depends on oxidation of glucose, glutamine, and fatty acids [5]. These contrasting metabolic strategies highlight plasticity in how to drive the oxidative metabolism-histone acetylation axis that boosts inducible gene expression during macrophage activation. In the CD40 activated macrophages, reliance on glutamine and fatty acids may be a metabolic adaptation to glucose limitation in the TME, although the extracellular release of lactate, which has immunosuppressive roles in this setting, could limit TAM anti-tumorigenic activities.

In summary, the study by Ho and colleagues elegantly defines a metabolic pathway, previously unknown in macrophages, in which fatty acids and glutamine conspire to promote pro-inflammatory and anti-tumorigenic activities in CD40-activated TAMs (Fig. 1).
